# Does Living Donor Hyperoxia Have an Impact on Kidney Graft Function After Transplantation?

**DOI:** 10.5812/numonthly.11870

**Published:** 2013-06-19

**Authors:** Zohreh Rostami, Behzad Einollahi, Mohammad Hassan Ghadiani

**Affiliations:** 1 Nephrology and Urology Research Center, Baqiyatallah University of Medical Sciences, Tehran, IR Iran; 2 Shahid Beheshti University of Medical Sciences, Tehran, IR Iran

**Keywords:** Oxygen Inhalation Therapy, Kidney, Transplantation, Delayed Graft Function

## Abstract

**Background:**

Improvement in the outcome of organ transplantation is related to advances in patient selection criteria, organ preservation, operative techniques, perioperative care and efficacy of immunosuppressive agents.

**Objectives:**

We aimed to evaluate the effects of higher levels of arterial PaO_2_ in donors on DGF (delayed graft function).

**Patients and Methods:**

Forty patients over 18 years old with stage 4-5 chronic kidney disease (CKD) who received a kidney from living donors were enrolled. They were randomly grouped in to the case (n = 17) and control (n = 23) groups and were followed for 2 weeks after transplantation. Donors were exposed to 60% oxygen for at least 2 hours with a face-mask (venture mask) for 2 consecutive days before transplantation until arterial oxygen pressure increased in arterial blood gas to 200 mmHg. Neutrophil gelatinase associated lipocalin (NGAL), Interleuk-18 (IL-18), tumor necrosis factor- α (TNF-α) and transforming growth factor–β (TGF-β) could be good biomarkers for early diagnosis of kidney injury in renal transplant recipients; we assessed kidney function with these biomarkers.

**Results:**

Forty living kidney transplantations including 17 cases and 23 controls were performed; female gender was more prevalent in recipients (n = 16, 40%). The mean age of recipients was 36.1 ± 12.4 (18-67) years old. DGF was detected in 2 (5.95%) individuals, from whom one was in the case group and the other one in the control group. In the univariate analysis, there was no significant correlation between age and biomarkers in urine and serum unless for the second serum NGAL (P = 0.02, r = -0.06) and second urine IL 18 (P = 0.03, r = -0.5) which had a negative correlation, and first urine TNF α (P = 0.02, r = 0.7) which had a positive correlation.

**Conclusions:**

Oxygen therapy in the case group had no significant impact on protection from DGF.

## 1. Background

Parallel to the rapid growth of the incidence of end stage renal disease (ESRD) (1), demand for kidney transplants has grown faster than the actual supply of kidneys (2). However, ESRD has been estimated to be between 15 and 80 per one million individuals (3). Deceased donor kidney transplants offer an additional possibility for patients requiring renal replacement therapy. Nevertheless, the improvement in the outcome of organ transplantation is related to advances in patient selection criteria, organ preservation, operative techniques, perioperative care and efficacy of immunosuppressive agents (4).

Delayed graft function (DGF) defined by the need for dialysis within the first week post-transplantation, portends a foreshortened allograft survival of 73% by the first year compared to 83% for recipients who do not require dialysis (5). The reported incidence of DGF from various centers ranges from 13.3 to 52 % (5). Organ preservation has an impact on allograft renal function (2). On the other hand, hyperbaric oxygen (HBO) therapy has been revealed to modulate both cellular and humoral immune response as well as lessen the severity of reperfusion injury (2). However, there is no evidence for the harmful effect of oxygen challenge via invasive mechanical ventilation on the risk of DGF in deceased donors before recovery of kidney.

## 2. Objectives

Although, oxygen is a vital substrate in the reduction of hypoxia, anoxia and ischemia, it also acts as a toxic metabolite due to reactive oxygen species (ROS) production (2, 3). On the other hand, elevated oxygen tension during reperfusion may actually promote necrosis (6).

Regarding this controversy, the authors attempted to evaluate the effects of higher levels of arterial PaO_2_ in donors on DGF. Also, this paper provides an overview of the relationship between oxygen therapy and DGF and the potential harmful impacts of oxygen therapy in kidney transplantation.

## 3. Patients and Methods

### 3.1. Participants

This cross-sectional study was initiated after the protocol was approval by the Baqiyatallah Medical University Ethics Committee and informed consent was obtained regularly from all recipients and donors between 2006 and 2007.

Forty patients over 18 years of age with stage 4-5 chronic kidney disease (CKD) who received a kidney from a living donor were enrolled. They were randomly grouped in to the case (n = 17) and control (n = 23) groups and were followed for 2 weeks after transplantation.

The recipients who had long ischemic time, vascular problems, hemodynamic instability during operation, history of smoking and diabetes were excluded. In addition, donors with past medical history of pnumothrax, asthem, seizures, optic neuritis, middle ear surgery and heavy smokers were excluded.

The immunosuppressive protocol was a triple therapy initiated by cyclosporine (6 mg/kg/day), mycophenolate mofetil (1 g/day) and prednisolone (2 mg/kg/day). As neutrophil gelatinase associated lipocalin (NGAL) (7, 8), interleuk-18 (IL-18), tumor necrosis factor-α (TNF-α), transforming growth factor-β (TGF-β) could be good biomarkers for early diagnosis of kidney injury in renal transplant recipients, we assessed kidney function with these biomarkers.

### 3.2. Oxygen Protocol

Donors were exposed to 60% oxygen for at least 2 hours with a face-mask (venture mask) for 2 consecutive days before transplantation until arterial oxygen pressure increased in arterial blood gas to 200 mmHg.

### 3.3. Variables

Following variables were gathered for all recipients: age, gender and urine NGAL, IL-18, TNF-α, TGF-β for consecutive days.

### 3.4. Definitions

DGF was defined as dialysis requirement in the first week after kidney transplantation (9).

### 3.5. Statistical Analysis

Data were analyzed with SPSS, version 17.0. Quantitative variables have been expressed as mean ± SD and qualitative variables have been shown by percentages. Continuous data were compared by Student’s t-test and categorical data were analyzed using the Chi-square or Fisher’s exact test. P values less than 0.05 were significant.

## 4. Results

### 4.1. General Characteristics

Forty living kidney transplantations including 17 cases and 23 controls were performed; female gender was more prevalent in recipients (n = 16, 40%). In the case group male: female ratio was 1:2.4 while in the control group, this was 1.3:1 (P = 0.1). The mean age of recipients was 36.1 ± 12.4 (18-67) years old. DGF was detected in 2 (5.95%) individuals, of whom one was in the case group and the other one in control group. Mean of serum and urine detected biomarkers are summarized in Table 1. Nonparametric correlation between age and urine or blood kidney injury biomarkers after transplantation in the case group is summarized in Table 2.

**Table 1.** Mean and Standard Deviation of Serum and Urine Kidney Injury Biomarkers During 4 Days

Biomarker	Mean ± SD (min-max)
**Urine NGAL 1^a^**	534.55 ± 460.8 (5-1190)
**Urine NGAL 2**	227.27 ± 366.001 (5-1215)
**Urine NGAL 3**	100.38 ± 187.4 (5-710)
**Urine NGAL 4**	124.17 ± 152.52 (25-430)
**Serum NGAL 1**	788.95 ± 535.19 (40-1900)
**Serum NGAL 2**	395.38 ± 438.35 (910-1940)
**Serum NGAL 3**	325.2 ± 230.9 (915-1010)
**Serum NGAL 4**	237.89 ± 225.55 (10-980)
**IL 18, 1^a^**	10.19 ± 1714.6 (0-5000)
**IL 18, 2**	1148.2 ± 1711.2 (0-5000)
**IL 18, 3**	1211.38 ± 1582.6 (115-5000)
**IL 18, 4**	1177.73 ± 1496.6 (0-5000)
**TNF α, 1^a^**	31.75 ± 37.8 (0-120)
**TNF α, 2**	96 ± 177.2 (10-680)
**TNF α, 3**	208.9 ± 313.74 (10-1000)
**TNF α, 4**	279.6 ± 313.9 (12-1000)
**TGF β, 1^a^**	4760 ± 3669.76 (0-12000)
**TGF β, 2**	4104.17 ± 2703.18 (470-9000)
**TGF β, 3**	2705.38 ± 2208.15 (0-8000)
**TGF β, 4**	3900.6 ± 2480.15 (220-8000)

^a^Abbreviations: NGAL, neutrophil gelatinase associated lipocalin; IL-18, Interleuk-18; TNF-α, tumor necrosis factor- α; TGF-β, transforming growth factor–β

**Table 2.** Nonparametric Correlation Between Age and Urine or Blood Kidney Injury Biomarkers After Transplantation in Case Group During 4 Days

	Correlation (r)	P
**Urine NGAL 1^a^**	0.08	0.8
**Urine NGAL 2**	0	1
**Urine NGAL 3**	0.4	0.7
**Urine NGAL 4**	-	-
**Serum NGAL 1**	0.3	0.4
**Serum NGAL 2**	-0.6	0.02
**Serum NGAL 3**	-0.03	0.9
**Serum NGAL 4**	-0.6	0.05
**IL 18, 1^a^**	-0.6	0.07
**IL 18, 2**	-0.5	0.03
**IL 18, 3**	-0.2	0.5
**IL 18, 4**	0.1	0.6
**TNF α, 1^a^**	0.7	0.02
**TNF α, 2**	0.6	0.07
**TNF α, 3**	0.5	0.6
**TNF α, 4**	0.03	0.9
**TGF β, 1^a^**	0.07	0.8
**TGF β, 2**	-0.7	0.1
**TGF β, 3**	-0.7	0.1
**TGF β, 4**	0.2	0.4

^a^Abbreviations: NGAL, neutrophil gelatinase associated lipocalin; IL-18, Interleuk-18; TNF-α, tumor necrosis factor- α; TGF-β, transforming growth factor–β

### 4.2. Univaritate Analysis

In the univariate analysis, there was no significant correlation between age and biomarkers in urine and serum unless for the second serum NGAL (P = 0.02, r = -0.06), second urine IL 18 (P = 0.03, r = -0.5) which had a negative correlation, and first urine TNF-α (P = 0.02, r = 0.7) which had a positive correlation. Oxygen therapy in the case group had no significant impact on protection from DGF.

## 5. Discussion

In the current study, we studied the effect of donor hyperoxia (PaO_2_ ≥ 200 mmHg) on kidney function after kidney transplantation. Despite the mentioned beneficial effects of hyperbaric oxygen (HBO), we revealed that donor hyperoxia, was not associated with advantages. Einollahi et al. in 2011 (4) showed that this level of hyperoxia resulted in 5 folds probability for DGF presentation compared to PaO_2 _less than 200 mmHg in deceased donor. According to the previous studies (3, 10, 11), hyperbaric oxygen exposure can preserve organs in a more optimal condition for transplantation. Although experimental studies have suggested that hyperoxia induced by hyperbaric oxygen may be favorable in the management of reperfusion injury, its mechanism remains unclear (10). However, there are also reports on the immunomodulatory effects of hyperbaric oxygen, which have been directed towards its effect on ischemia reperfusion injury (IRI) (12, 13). Although researchers have also demonstrated a suppressive effect on both humoral and cell mediated immunity in animal studies (3) as well as alternation in cell surface MHC class I antigen expression (3, 14), yet HBO has been noted to increase ROS production (15, 16).

Moreover, the deteriorative outcome of pre-operation hyperoxia on DGF may be described by the toxic impact of oxygen-free radicals released through reperfusion of the ischemic region (17). Oxygen-free radicals participate in lipid peroxidation and lead to endothelium and cell membrane injury, ensuing in a total defect of cellular integrity. On the other hand, cell cannot be metabolized aerobically anymore which leads to a progressive energy reduction and cytosolic Ca^++^ overload. Reperfusion of the graft in the presence of decreased cellular energy may prone further damage resulting in cell death and possible renal allograft failure (10). In addition, it seems that reperfusion not only leads to energy deficiency within the kidney and endothelial cells as well as the products of reactive oxygen species (ROS) but also promotes release of potent inflammatory cytokines such as tumor necrosis factor-α (TNF-α) and interleukin-1 (IL-1) (18) as well as factors such as the pro-apoptotic tumor protein p53 gene (TP53) and the anti-apoptotic, antioxidant heme oxygenase 1 gene (HMOX1) that play an important role in modulating apoptosis. Though in acute kidney injury the TNF-α gene, transforming growth factor β1 gene (TGF-β1), and interleukin 10 gene (IL-10) have more important effects on regulation of inflammatory response (5). In kidney allograft, cell adhesion molecules are expressed by TNF-α and promote kidney damage (19-21). On the other hand, there is greater expression of TNF-α in kidney allografts with DGF than those without DGF (5). Therefore, donor hyperoxia that enhances TNF-α activity could predispose recipients to DGF (11). On the basis of previous studies, although HBO preserves tissue oxygenation but immunomodulatory effect of HBO therapy has been attributed predominantly to the hyperbaric effect rather than to hyperoxia (3, 22). Animal studies have substantiated an inhibitory effect of HBO therapy in the absence of hyperoxia on IFN-γ. Subsequently, despite of the increase in oxygen tension, hyperbaric oxygen affects cellular cytoskeleton, leading to a reduction in IFN-γ. In other words mechanism of HBO immunomodulation is different from energy formation (3).

The other mechanism of this toxic effect of HBO might be associated with lipid peroxidation which, cause cell membrane and organelle damage in the IRI state and level of injury augments during exposure to normobaric 100% oxygen (3, 15, 16). Therefore, it seems that oxygen saturation is more important than oxygen pressure in organ damage and DGF induction.

Limitations: it is possible that the association of kidney injury with other predictors were not seen in our study. The little sample size is the most important limitation in our study.

Conclusion: The current study revealed that normobaric hyperoxia of living donors before kidney transplantation has no effect on kidney function in renal transplant recipients. Further studies are required to confirm our findings.
